# Interaction of Nanomaterials with Cells and Tissues

**DOI:** 10.3390/ijms241713667

**Published:** 2023-09-04

**Authors:** Peter Veranič, Igor Križaj

**Affiliations:** 1 Institute of Cell Biology, Faculty of Medicine, University of Ljubljana, SI-1000 Ljubljana, Slovenia; 2 Department of Molecular and Biomedical Sciences, Jožef Stefan Institute, SI-1000 Ljubljana, Slovenia

## 1. Introduction

Nanomaterials have gained enormous importance in biomedicine in recent years, both in basic and applied sciences. The distinctiveness of these particles, which has driven their investigation and general use, is that due to their small size and thus high surface-to-volume ratio, they acquire unique properties when compared to the same material with a larger volume. As such, nanomaterials can more intensively interact with living cells and tissues. Due to the massive production and extensive use of these materials, we are constantly exposed to them via food, cosmetics, and in the manufacture of virtually all types of industrial products. The long-term accumulation of nanomaterials in tissues could lead to certain medical problems that have yet to be thoroughly studied. For example, TiO_2_ nanoparticles (NPs) can induce inflammation by increasing levels of NF-κB, IFN-γ, TNF-α, NO, and many other signalling molecules [1]. On the other hand, nanomaterials have recently been used in medicine as an advanced system for cancer treatment [2], drug/gene delivery [3], and tissue engineering [4]. They were successfully employed also in the rapidly developing field of nano-sensors and in bio-imaging [5]. In this Special Issue, a series of selected original and review papers present new findings about the interactions of nanomaterials with the cells and tissues of predominantly mammalian organisms.

## 2. Commentary on the Contents of the Special Issue

Since TiO_2_ NPs are among the most commonly used NPs in commercially available products, including cosmetics, sunscreens, paints, food and packaging, photoactive cement, and innovative textiles [6,7], Miu et al. [8] investigated the cytotoxic and genotoxic effects of P25 TiO_2_ NPs and the possible modulation of its toxicity by impregnating it with small amounts of iron and nitrogen atoms. Although the phototoxicity of P25 TiO_2_ NPs is proportionally increased by 1 to 10% iron doping because of the generation of oxidants via the Fenton reaction [9], the authors noted an attenuation of the toxic effects of iron-doped P25 TiO_2_ NPs on lung fibroblasts, possibly due to their generation of some reactive species in the first hours of exposure, which induced the antioxidant mechanisms and enabled fibroblasts to counteract excessive oxidation by P25 TiO_2_ NPs.

In their original article, Pavlin at al. [10] compared the importance of NPs’ protein corona composition as well as their physicochemical composition for the response of immune cells. In these contexts, they examined the response of THP-1 macrophages to different types of NPs, focusing on cell viability, reactive oxygen species (ROS) production, and cytokine secretion. All TiO_2_ and SiO_2_ NPs tested exhibited moderate toxicity and a transient inflammatory response as evidenced by an increase in ROS, and IL-8 and/or IL-1 cytokine secretion. Correlation analysis showed that specific NP corona proteins were associated with the induction of cytokine secretion.

Gojznikar et al. [11] presented a review on the positive and negative effects of the potentially toxic photocatalytic TiO_2_ NPs properties on eukaryotic cells of both mammalian and non-mammalian origin. There is evidence that if ingested, TiO_2_ NPs can lead to inflammation of the colon mucosa and induce apoptosis, necrosis, and necroptosis in human and other eukaryotic cells [12]. TiO_2_ NPs enter the organism mainly by inhalation, ingestion as a component of a dietary supplement (E171), or penetration through the skin via TiO_2_ NP-containing cosmetic products and pharmaceuticals [13]. On the other hand, the production of ROS induced by TiO_2_ NPs upon its exposure to UV light could be used for the photodynamic therapy of cancer [14]; the ROS induced by illuminated TiO_2_ NPs can induce cancer cell death and thus inhibit tumour growth. Wang et al. [15] reported that combined exposure to TiO_2_ NPs and treatment with UV A resulted in a significant increase in the extent of necrotic areas and apoptosis indices in mice with gliomas. The effects of TiO_2_ NPs on eukaryotic cells [16] are highly multiplied by the flow of materials in ecosystems. Its concentration along the food chain makes TiO_2_ NPs an important potential toxicant, especially due to its ever-growing number of potential applications. TiO_2_ NPs clearly possess several properties that can be beneficial, but also detrimental, to eukaryotic cells.

Another type of nanomaterials that are widely used are carbon-based nanomaterials. Their properties and interactions with human cells were studied by Svadlakova et al. [17]. They focused on the immunotoxic potential of carbon-based nanomaterials, especially in professional phagocytes since they play a central role in the processing and elimination of foreign particles. Carbon-based nanomaterials represent a group heterogeneous inorganic nanomaterials, such as amorphous particles, i.e., ultrafine carbon particles; carbon NPs; and carbon dots; as well as sp^2^ (nanotubes, graphene, fullerenes, carbon quantum dots) and sp^3^ carbon allotropes, such as nanodiamonds [18]. Depending on the physicochemical properties of carbon-based nanomaterials, in an attempt to engulf and process these NPs by phagocytes, a pro- or anti-inflammatory response can be induced.

A specific use of nanodiamonds as a special carbon-based nanomaterial in urology was presented in a review paper by Zupančič and Veranič [19]. They focused on the potential use of nanodiamonds in two important urologic diseases: bladder cancer and bacterial cystitis. In the treatment of bladder cancer, nanodiamonds can be used as effective carriers for various types of anticancer drugs; such as cytostatics, which decrease the proliferation of cancer cells and induce their apoptosis; and retinoic acid, which stimulates the differentiation of urothelial cells and prevents the recurrence of bladder cancer [20]. The drug internalisation of NPs caused by endocytosis is the most efficient way to load cancer cells with cancer-specific drugs. Recently, endocytosis was found to be considerably more active in urothelial carcinoma cells in comparison to the normal urothelial cells. This allows for the highly selective targeting of cancer cells [21]. The specific and very rare property of nanodiamonds to penetrate into superficial urothelial cells allows for the additional use of these NPs in the treatment of the most common urological problem, chronic bacterial cystitis, which is rarely successful by using antibiotics alone. Treatment with antibiotics bound to nanodiamonds results in better penetration of the drug into urothelial cells, where it concentrates in the cytosol to act on the intracellular pools of uropathogenic bacteria more efficiently [22]. In addition, nanodiamonds can be coated with mannose, which is commonly used to prevent uropathogenic Escherichia coli from adhering to the urothelium and even inhibit the growth of bacterial biofilms that protect bacterial communities [23].

Due to potentially toxic side effects of NPs, it is necessary to consider the protection of cells and tissues upon administering the NP-drug conjugate therapy. An innovative approach in this respect was studied by Repar et al. [24]. They described the efficacy of oleic acid to protect endothelial cells from silica-coated superparamagnetic iron oxide NPs (SPIONs). Oleic acids are considered to be highly resistant to oxidation and are able to enhance the activity of antioxidants, such as tocopherols [25]. Oleic acids have also been found to have a protective effect against cytotoxicity induced by oxidative stress triggers [26]. Due to their superparamagnetic properties, SPIONs have a large potential in medicine, as magnetic drug carriers, for the hyperthermic treatment of cells, as contrast agents in magnetic resonance imaging, and for imaging with superparamagnetic NPs [27]. SPIONs have been considered as biocompatible, but recent studies suggested that they in fact might cause aberrant cellular responses, such as DNA damage, oxidative stress, mitochondrial membrane dysfunction, changes in gene expression, and lipid peroxidation [28]. The results presented by Repar et al. [24] show that exogenously added oleic acids can protect endothelial cells from SPION-induced oxidative stress and cell death.

## 3. Conclusions

This Special Issue highlights the extremely interesting topic of NPs—their bright and shady sides. Through selected papers, their potential in various biomedical applications has been pointed out. The inspiring perspective of the field has been emphasized by presenting new applications of NPs in medicine, for example the use of nanodiamonds in bladder cancer and bacterial cystitis treatments. Threats concerning potential toxicity of these new materials have not been overlooked. Rather, they have been exposed by showing that NPs can induce serious inflammation and trigger diseases, such as cancer and neurodegenerative diseases. Encouragingly, effective protective solutions are being developed. As presented in our Special Issue, oleic acid and the impregnation of NPs with iron or nitrogen atoms have been demonstrated to significantly mitigate side effects inflicted by various types of NPs.
